# Intestinal Diseases of Children

**Published:** 1890-10

**Authors:** 


### Book Reviews.

Intestinal Diseases of Children, volume 1, by A. Jacobi, M. D., Clinical Professor of Diseases of Children in College of Physicians and Surgeons, New York. Geo. S. Davis, publisher.

We have read with no little interest and pleasure the above named volume. The wide-spread distinction of the author in this department of medicine is alone sufficient to ensure it no mean place in the literature of the day, while the subject matter of discussion renders it a work of great practical importance, and a valuable addition to the "Leisure Library" of the busy practitioner. The time is ripe for maturer consideration of infant feeding, its errors and effects. In fact, no question deserves more careful and attentive study than this fruitful source of disease among infants. Hence we welcome this timely written treatise, which, though brief in whole, yet elaborate in detail, affords much aid in the selection of proper food for the various pathological, as well as physiological, conditions of the digestive system.

J. C. J.

---

EXPLANATORY.—Owing to delays in getting engaged matter, THE JOURNAL is forced to apologize for late issue. An ad. reading notice appears on preceding page—printer's mistake—too late to change. Efforts to keep from leaving blank spaces on certain pages will continue, and we hope to have every page full—as desired.

